# Editorial: Non-Standard Work, Self-Employment and Precariousness

**DOI:** 10.3389/fsoc.2020.00063

**Published:** 2021-03-15

**Authors:** Valeria Pulignano, Annalisa Murgia, Emiliana Armano, Marco Briziarelli

**Affiliations:** ^1^KU Leuven, Leuven, Belgium; ^2^Department of Social and Political Sciences, University of Milan, Milan, Italy; ^3^University of New Mexico, Albuquerque, NM, United States

**Keywords:** non-standard work, self-employment, subjectivities, precariousness, social exclusion, hybrid areas of work, social protection

The increased level of insecurity in labor markets has generated much debate on precarious work arrangements—from illegal and temporary work to home working, piecework, freelancing, and online jobs—based on the assumption that the ongoing deregulation and transition to flexible labor markets incur higher risks for the labor force (Eichhorst and Marx, 2015; Pulignano, 2018). Situations of precariousness are measured by the extent to which the emerging work arrangements impact on the stability of employment and the access to social protections (Kalleberg, 2018). With the aim to analyze the social consequences of labor market flexibilization, and to gain better understanding of non-standard work arrangements (Bosch, 2004), more attention is needed on the heterogeneous labor market statuses and types of contracts that are different from what has so far been considered a standard employment relationship. Labor market transformations over time have in fact blurred the differences between the main categories traditionally used to interpret work and employment, eroding the usefulness of concepts such as “standard” and “non-standard,” and even blurring the distinction between the statuses of self-employed workers and waged employee.

The proliferating of new and old risks for workers with non-standard forms of employment, including those in a hybrid position between autonomous and dependent work, poses relevant questions for those who are interested in labor market transformations: What are the relations between non-standard and hybrid forms of employment and situations of precarious work? How these work arrangements differ across national contexts in terms of employment protection and workers' rights? What are the main differences and similarities in terms of class, migrant status, gender and age? How are work identities constructed to create new and hybrid types of workers? Under what conditions are these workers able to develop forms of collective representation? How can the collective representation and practices of organizing be articulated, and how do they manage to be widespread and effective?

The goal of this Research Topic is to share innovative theoretical and methodological lenses able to deconstruct what we still call — by difference — “non-standard” or “a-typical” work. In fact, although criticized by many, the current definitions are still anchored in the categories created *ad hoc* to interpret the Fordist model. To define the emerging work arrangements, and to understand to what extent they produce situations of precariousness, innovative approaches are required, that can only be built through the dialogue between different theoretical and methodological perspectives, able to grasp the new forms of work and employment and the connected risks of precariousness and social exclusion.

## Author Contributions

All authors listed have made a substantial, direct and intellectual contribution to the work, and approved it for publication.

## Conflict of Interest

The authors declare that the research was conducted in the absence of any commercial or financial relationships that could be construed as a potential conflict of interest.
